# Insight into implementation of facility-based integrated management of childhood illness strategy in a rural district of Sindh, Pakistan

**DOI:** 10.3402/gha.v6i0.20086

**Published:** 2013-07-05

**Authors:** Nousheen Akber Pradhan, Narjis Rizvi, Neelofar Sami, Xaher Gul

**Affiliations:** 1 Department of Community Health Sciences, Aga Khan University, Karachi, Pakistan; 2 Willows Foundation, Karachi, Pakistan

**Keywords:** IMCI, IMCI Pakistan, child health, under-five mortality, under-five morbidity, primary health care in children under five, IMCI implementation barriers and supports

## Abstract

**Background:**

Integrated management of childhood illnesses (IMCI) strategy has been proven to improve health outcomes in children under 5 years of age. Pakistan, despite being in the late implementation phase of the strategy, continues to report high under-five mortality due to pneumonia, diarrhea, measles, and malnutrition – the main targets of the strategy.

**Objective:**

The study determines the factors influencing IMCI implementation at public-sector primary health care (PHC) facilities in Matiari district, Sindh, Pakistan.

**Design:**

An exploratory qualitative study with an embedded quantitative strand was conducted. The qualitative part included 16 in-depth interviews (IDIs) with stakeholders which included planners and policy makers at a provincial level (*n*=5), implementers and managers at a district level (*n*=3), and IMCI-trained physicians posted at PHC facilities (*n*=8). Quantitative part included PHC facility survey (*n*=16) utilizing WHO health facility assessment tool to assess availability of IMCI essential drugs, supplies, and equipments. Qualitative content analysis was used to interpret the textual information, whereas descriptive frequencies were calculated for health facility survey data.

**Results:**

The major factors reported to enhance IMCI implementation were knowledge and perception about the strategy and need for separate clinic for children aged under 5 years as potential support factors. The latter can facilitate in strategy implementation through allocated workforce and required equipments and supplies. Constraint factors mainly included lack of clear understanding of the strategy, poor planning for IMCI implementation, ambiguity in defined roles and responsibilities among stakeholders, and insufficient essential supplies and drugs at PHC centers. The latter was further substantiated through health facilities’ survey findings, which indicated that none of the facilities had 100% stock of essential supplies and drugs. Only one out of all 16 surveyed facilities had 75% of the total supplies, while 4 out of 16 facilities had 56% of the required IMCI drug stock. The mean availability of supplies ranged from 36.6 to 66%, while the mean availability of drugs ranged from 45.8 to 56.7%.

**Conclusion:**

Our findings indicate that the Matiari district has sound implementation potential; however, bottlenecks at health care facility and at health care management level have badly constrained the implementation process. An interdependency exists among the constraining factors, such as lack of sound planning resulting in unclear understanding of the strategy; leading to ambiguous roles and responsibilities among stakeholders which manifest as inadequate availability of supplies and drugs at PHC facilities. Addressing these barriers is likely to have a cumulative effect on facilitating IMCI implementation. On the basis of these findings, we recommend that the provincial Ministry of Health (MoH) and provincial Maternal Neonatal and Child Health (MNCH) program jointly assess the situation and streamline IMCI implementation in the district through sound planning, training, supervision, and logistic support.

Globally, the five major preventable causes of child deaths are acute respiratory infections (including pneumonia), diarrhea, measles, malaria, and malnutrition (1). Of the 6.9 million under-five deaths which occurred in 2011, two-thirds were attributable to pneumonia, diarrhea, measles, and few other causes (2). WHO and UNICEF launched integrated management of childhood illnesses (IMCI) in the 1990s to reduce morbidity and mortality associated with these preventable conditions (3). IMCI offers a holistic approach to manage childhood morbidity; it has three components including case management training, strengthening of the health system, and improvement of family and community practices for child health and development (3).

To date, over 113 countries around the world have introduced IMCI strategy in their health systems (4). At the downstream level, training of health workers in IMCI has shown to improve the quality of service delivery in terms of improved assessment and management of sick children (5, 6). Adequate availability of essential drugs and supplies is also one of the critical components for the practice of IMCI algorithm by trained staff (7). At the upstream level, advocacy for IMCI and strong district capacity with a clear definition of roles have also received attention as determinants of IMCI implementation (4).

A multi-country evaluation conducted in 2002 and various countries’ experiences with IMCI implementation has demonstrated that many countries lack adequate health system support for IMCI implementation including poor adherence to IMCI guidelines, high turnover among trained staff, inadequate supervision, and insufficient availability of drugs, equipments, and referral facilities (8–11). Moreover, poor political will for strategy implementation, IMCI being viewed as a vertical program, lack of integration with health management information systems, and absence of IMCI policy statement from the Ministry of Health (MoH) have also been identified as key policy-level constraints (12).

Pakistan is a developing country with a population of 190 million (13). In 2011, Pakistan's MoH was devolved to the provinces under a constitutional amendment (14). The existing health care system is based on a primary health care (PHC) model having three tiers: the first-level facilities are Basic Health Units (BHUs), Rural Health Centers (RHCs), and Maternal and Child Health (MCH) Centers; secondary-level facilities are Tehsil Headquarter Hospitals (THQs) and District Headquarter Hospitals (DHQs); and third-level facilities are tertiary and teaching hospitals (15). Over the last decade, Pakistan's high infant and under-five mortality rates, that is 78 and 94 per 1,000 live births, respectively, have remained more or less stagnant with diarrhea, pneumonia, malnutrition, and measles being the major killers of children under 5 years old (16).

Pakistan endorsed IMCI strategy in 1998 (17). However, given the high neonatal mortality in Pakistan, the strategy was modified to include management of neonatal illnesses (18). District-level implementation was launched in 2000 in two districts of Punjab province and since then it has spread out across Pakistan with wide variations between provinces (17). Evaluation of the early implementation phase of IMCI in 2002 demonstrated several strengths; for instance health workers had adequate knowledge about first-line drugs for the management of various diseases and gave detailed instructions to caretakers’ for follow-up visit. However, some weaknesses, such as irregular supervision, shortage of IMCI-related drugs and equipments, and an inefficient referral system, were also identified (19). Later, IMCI was scaled-up by the MoH with the support of various partners including the National Maternal Neonatal and Child Health (NMNCH) program (17).

The country is currently in the expansion phase of IMCI and by 2011, 95 out of 135 districts had started the implementation of IMCI (20). Since the initial process evaluation in 2002, to the best of our knowledge, the implementation of this important strategy has not been appraised. While outcomes are critical to assess program's effectiveness, an assessment of processes provides key information regarding supports and constraints that influence effective implementation. Therefore, the objective of our study was to determine the factors influencing facility-based IMCI implementation at public-sector PHC facilities in a district of Sindh province in Pakistan.

## Methods

### Study design

**Fig. 1:**
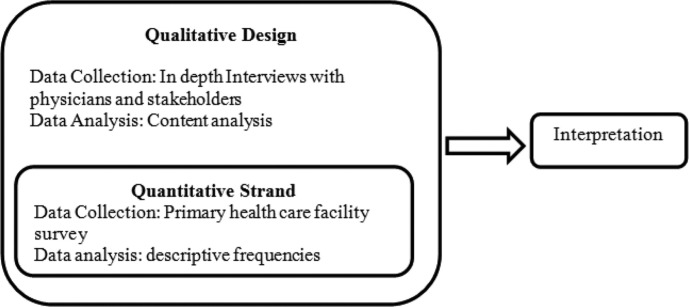
Embedded design.

This was an exploratory qualitative study with an embedded quantitative strand (Fig. 1) to enhance the overall research design (21). The qualitative part of the study included in-depth interviews (IDIs) with IMCI-trained physicians at PHC level, implementers, and managers at the district level, and planners and policy makers at the provincial level. The quantitative strand was integrated within the qualitative design whereby PHC facilities served by IMCI-trained physicians were surveyed to assess the availability of essential IMCI supplies and drug. The facility survey was conducted concurrently with in-depth physician interviews. The integration of quantitative strand within qualitative design facilitated during the analysis phase by triangulating the respondent's views about logistic support (availability of essential drugs and supplies) with the findings obtained from the PHC facility survey.

### Study site

The study was conducted in a rural district Matiari of Sindh province in Pakistan (Table 1). The district was selected for two main reasons: first, it is among the first districts in Sindh province to initiate IMCI implementation in 2006 (22) and second, Matiari allows easy access to rural PHC service providers as well as district-level stakeholders (implementers and decision makers). The study was approved by the Ethical Review Committee of The Aga Khan University, Karachi, Pakistan. Administrative approval was also obtained from the Executive District Officer-Health (EDO-H), Matiari district.

**Table 1:** Profile of the Matiari district (year 2009)

Total population	661,000
Total area	1,491 km^2^
Major occupation	Agriculture
Total union councils	19
Total thesils	3
Total health facilities	44
Taluka hospitals	3
Rural Health Centers (RHCs)	5
Basic Health Units (BHUs)	20
Government dispensaries	15
Maternal and Child Health Center (MCH)	1
(Information received from EDO-H Office Matiari)	

### Data collection

#### In-depth interviews

Stakeholders at the provincial level included key personnel involved in planning and policy making, whereas at the district level they were largely involved in implementation of health care programs and IMCI-trained physicians working at PHC facilities. Mapping of these stakeholders at both district and provincial levels was carried out to purposively involve key planners and implementers directly involved in planning, decision making, and routine management of IMCI-based service delivery. After a careful mapping exercise, purposive sampling was employed to select five provincial and three district stakeholders. Altogether, 16 IDIs were conducted with stakeholders at both district level (*n*=3) and provincial level (*n*=5) as well as with IMCI-trained physicians (*n*=8) deputed and providing services at PHC facilities in the district, where the health facility survey was conducted. Table 2 shows the characteristics of the respondents. See Appendix 1 for an abridged version of the interview guide.

**Table 2:** Characteristics of study respondents

Characteristics	Stakeholders^1^ (*N*=8)	Trained physicians (*N*=8)
Age		
30–35	2	2
35–40	2	1
40–45	4	5
Sex		
Male	7	6
Female	1	2
Years of experience		
1–3	3	2
3–5	2	4
>5 years	3	2
Training in IMCI		
Trained	3	8
Untrained	5	0

1 Stakeholders at provincial level included, key personnel involved in planning and policy making (decision makers), whereas at district level it included personnel involved in implementation of health care programs (implementers).

The objective of the interviews was to gain insight to factors supporting and constraining IMCI implementation in the district. Exclusion criteria for participants were defined pragmatically and included unavailability of key personnel and IMCI-trained physicians and consent refusal from study participants. Recruitment of physicians for IDIs continued until data saturation was achieved, that is when no new data appears in interviews and there was sufficient depth of information obtained on factors influencing IMCI implementation from trained doctors (23). Separate semi-structured interview guides were used for both groups of respondents. The semi-structure guide developed for interviews with physicians mainly focused on understanding about the strategy, positive and negative factors influencing their practice of the strategy, and their perception of monitoring and supervision for trained doctors. Whereas, semi-structured guide for stakeholders mainly revolved around knowledge about the strategy, policy, and planning initiatives in implementation, expected responsibility in implementation and perception about implementation of the strategy at PHC facilities. Probes were used in both semi-structured guides to explore respondents’ responses. Interviews were conducted after verbal and written consent. Individual interviews lasted between 60 and 90 min and all were audiotaped with participants’ permission. Venues for stakeholders’ interviews were at their designated offices in Matiari, Hyderabad, and Karachi cities. Interviews with physicians were conducted at their respective PHC facilities. Preference of the language used during interviews was at respondents’ discretion. Both English and Urdu languages were used during interviews with stakeholders, while interviews with physicians were conducted in Urdu. Confidentiality was maintained during data collection by keeping the recorded and documented information in safe folders with access limited to the principal investigator. All the interviews were then transcribed verbatim within 2 weeks and were translated into English where required and reviewed by the principal investigator (PI) for accuracy. Anonymity was maintained by removing all personal identifiers from the transcripts, if present. No compensation, in any form, was offered to the study respondents.

#### Primary health facility survey

Health facility survey was designed to assess the availability of IMCI-related logistics and supplies (essential equipments, supplies, and drugs) at PHC facilities in the district. For this purpose, WHO's facility survey checklist for the module: ‘Supplies, Equipment and Drug Availability’ was modified and adapted (24). Section 1 is composed of 30 IMCI-related equipments and supply items. Section 2 is composed of 27 essential IMCI drugs. Inclusion criteria included PHC facilities with at least one trained IMCI physician and consent given by the health facility manager. In total, 16 PHC facilities (eight BHUs, five RHCs, and three government dispensaries) met the criteria and were included in the survey after informed written consent by facility managers. The survey checklist was pre-tested in a public health care facility in Karachi. A data collector was recruited to assist in the survey and was trained and supervised in the field. The health facility survey was conducted concurrently with IDIs.

### Analysis

Data analysis was carried out after data collection of qualitative and quantitative strands were completed. The data from health facility survey were entered in Epi Data and later imported to SPSS Version 15.0 for analysis. Qualitative content analysis was used to interpret the manifest content (what the text says) and latent content (interpretation of the underlying meaning of the text) (25). Interviews were read several times to develop an understanding of the respondents’ perception of factors influencing IMCI implementation, which constituted the unit of analysis. The text was divided into ‘meaning units’ that were condensed and labeled with a ‘code’ by the first author without losing the context (25) (Table 3). Codes were subsequently analyzed and grouped into nine categories to capture the manifest meaning. In the final step, two major themes, that is factors supporting and factors constraining IMCI implementation, were identified in consultation with co-authors. In the latent analysis, focus was on discovering the underlying meaning of the words or content gathered during the manifest part (25–27) thus giving an account of how IMCI implementation has received support and been constrained in Matiari district.

**Table 3:** An example of the analysis method

Analyzing units	Condensed analyzing units	Codes
If there is one designated provider in an isolated clinic, he will be able to give better attention for providing care to under-five-year-old children	Need of separate designated provider for better management	Designated providerBetter management
I am not told what I can do, how to monitor IMCI implementation; still not clear what is my job description specific to strategy …	Lack of clarity about IMCI implementation	Unclear role
For trained doctors, the system does not even have reporting forms and supplies. If you can't implement, you don't practice, you forget and you don't deliver.	Unavailability of reporting forms and supplies	Absence of supplies

Findings obtained from the facility survey concerning the availability of supplies, equipments, and drugs were triangulated with the relevant responses captured during IDIs.

## Findings

### Health facility survey

Table 4 lists the availability of 30 essential equipments and supplies including, OPD supplies, documentation tools, and vaccination supplies in three different types of PHC facilities. Findings indicated that with the exception of three items (weighing scale, thermometer, and clean water supply) none of the facilities had a complete stock of essential supplies and equipments. The highest stock recorded was 75% of supplies (15 out of 20 items) in only one facility. Mother counseling cards and towels for tepid sponging were not available in all surveyed facilities. IMCI recording tools were found to be available in the majority of the facilities but overall availability of supplies seemed to be inadequate in PHC facilities.

**Table 4:** Availability of essential equipments and supplies at PHC facilities

S. no.	Items	BHUs (*n*=8) (%)	RHCs (*n*=5) (%)	Govt. dispensaries (*n*=3) (%)	Total (*n*=16) (%)
1	Weighing scale 1–5 years	100	100	100	100
2	Weighing scale for baby (>1 year)	87.5	100	100	93.8
3	Supplies to mix ORS (cups and spoons)	0	20	0	6.3
4	Source of clean water	100	100	100	100
5	Thermometer	100	100	100	100
6	Towel (tepid sponge)	0	0	0	0
7	NG tube	0	20	0	6.3
8	IV infusion set	87.5	100	33.3	81.3
9	IV cannula (22–24 G)	87.5	100	33.3	81.3
10	Intramuscular syringe (5 cc)	75	60	100	75
11	Small-sized syringe (1–2 cc)	0	40	0	12.5
12	Nebulizer mask	37.5	60	0	37.5
13	Oxygen cylinder	37.5	80	0	43.8
14	Stock cards/drug log book	75	80	100	81.3
15	Mother's counseling cards	0	0	0	0
16	IMCI chart booklet/ wall charts	50	40	33.3	43.8
17	IMCI recording forms (under 2 months)	62.5	80	0	56.3
18	IMCI recording forms (2 months to 5 years)	75	80	0	62.5
19	Referral forms/slips	75	60	33.3	62.5
20	Growth cards	62.5	100	0	62.5
					
	Vaccination supplies				
	
	Items	BHUs (*n*=8)	RHCs (*n*=5)		Total (*n=*12)*
	
21	Vaccination cards	75	100		83.3
22	Vaccination register	87.5	100		91.7
23	Appropriate needles and syringes for all vaccines	87.5	100		91.7
24	Danger/safety box	87.5	100		91.7
25	Functioning fridge	87.5	100		91.7
26	Ice packs	87.5	100		91.7
27	Polio vaccines	87.5	100		91.7
28	BCG	62.5	100		75
29	Pentavalent	87.5	100		91.7
30	Measles	25	100		50

* Excluding government dispensaries which do not have mandate for vaccination supplies and one RHC which was found to be closed on the day of visit and on subsequent visit.

The mean availability of supplies in BHUs, RHCs, and government dispensaries is 55.6, 66, and 36.6%, respectively. It was noticed that facilities with a mandate for vaccination (*n=*12) scored high in terms of availability of vaccination supplies, for example, all surveyed RHCs had 100% of vaccination supplies. BHUs, however, had insufficient supply of BCG and measles vaccines with one facility reporting expired measles vaccine in the stock.

Table 5 highlights availability of 27 IMCI essential drugs (alongside few diluting agents) in three types of PHC facilities. Mean availability of drugs in BHU, RHCs, and government dispensary is 50.4, 45.1, and 56.7%, respectively.

**Table 5:** Availability of IMCI drugs at PHC facilities

S. no.	Categories	BHUs (*n*=8) (%)	RHCs (*n*=5) (%)	Govt. dispensaries (*n*=3) (%)	Total (*n*=16) (%)
1	ORS packets	100	40	100	81.3
2	First-line antibiotic for pneumonia and ear infection: Cotrimoxazole	75	80	100	81.3
3	Second-line antibiotic for pneumonia and ear infection: Amoxicillin	75	60	100	75
4	First-line antibiotic for dysentery and cholera: Nalidixic acid	0	0	0	0
5	Second-line antibiotic for dysentery: Metronidazole	100	100	100	100
6	Second-line antibiotic for cholera: Chloramphenicol	12.5	0	33.3	12.5
7	First-line antibiotic for malaria: Cholorquine	100	40	100	81.3
8	Second-line antibiotic for malaria: Sulfadoxine pyimethamine	75	20	100	62.5
9	Antibiotic for eye infection: Cholramphenicol ointment	0	0	0	0
10	Antibiotic for streptococcal sore throat: Benzathine penicillin or amoxicillin	87.5	80	100	87.5
11	Antibiotic for PSBI* Ampicillin or benzyl penicillcin and gentamicin	12.5	40	0	18.8
12	Bronchodilator for wheezing: Nebulized salbutamol	12.5	0	0	6.3
13	Bronchodilator for fast breathing: Oral salbutamol	87.5	100	100	93.8
14	Paracetamol	100	80	100	93.8
15	Vitamin A	0	0	0	0
16	Multivitamin/mineral supplements	100	60	66.7	81.3
17	Iron or folate tablets (ferrous sulphate)	75	20	0	6.3
18	Iron syrup (ferrous fumarate)	0	20	0	6.3
19	Deworming drug: Pyrantel Pamote	0	0	0	0
20	Gentian violet	0	0	33.3	6.3
21	Normal saline	87.5	100	100	93.8
22	Dextrose water	75	100	100	87.5
Pre-referral drugs					
23	Urgent referral: Cholramphenicol	0	0	0	0
24	Sever Malaria: Quinine	0	20	0	6.3
25	Dehydration: Ringers Lactate	87.5	100	100	93.8
26	Convulsions: Diazepam or Paraldehyde	37.5	60	66.7	50
27	Sterile water for injection	62.5	80	33.3	62.5

* possible severe bacterial infection

A few drugs such as ORS packets, antibiotic for pneumonia and ear infection, antibiotic for dysentery, bronchodilators for fast breathing, anti-pyretic, multi-vitamin supplements, and normal saline were available in majority of the facilities. It was noted that recommended drugs such as first-line antibiotics for dysentery and cholera, antibiotics for eye infection, drugs for deworming, vitamin A, and pre-referral drugs (chloramphenicol) were found to be missing from all types of facilities. Furthermore, only one RHC showed availability of 19 of the 27 listed drugs, whereas 15 of the 27 listed drugs were found to be available in only 25% (4 out of 16) facilities surveyed, which includes three BHUs and one government dispensary. The presence of substitute drugs was also noted as the majority of facilities used albendazole and mebendazole as the substitute drugs for pyrantel pamoate for deworming and gentamicin drops in place of chloramphenicol ointment for the treatment of eye infections.

### In-depth interviews

Two underlying themes emerged during the analysis: ‘factors supporting IMCI implementation’ and ‘factors constraining IMCI implementation’. These are described below in categories and presented in Table 6. The term ‘both group of respondents’ (which refers to physicians and planners/implementers) is stated in findings where necessary.

**Table 6:** Themes, categories, and codes describing the perceptions by stakeholders and physicians concerning factors influencing IMCI implementation in Matiari district, Sindh, Pakistan

Themes	Categories	Codes
Factors supporting IMCI implementation	Knowledge and perception about IMCI strategy Separate under-five clinic at PHC facilities	Holistic approach Practical and useful Significant strategy Improved child health Best option Facilitate implementation Designated provider Better attention Better management
Factors constraining IMCI implementation	Lack of clear understanding of IMCI strategy Lack of planning for IMCI implementation Ambiguity in roles and responsibilities toward IMCI implementation IMCI – a time-consuming task Emphasis on training vs. logistic support Lack of monitoring and supervisory mechanism Inefficient referral system	Unclear understanding Program for infants only Lack of knowledge Unclear with objective No planning Emphasis on training Poor planning Non-involvement of pediatricians Uncertainty Communication gap Lacked awareness Unclear role No guidance received No job description Helplessness Emphasized MNCH program to assist Non-involvement How to monitor What to do Increase patient flow Problematic to practice More time needed More documentation Disagreement by stakeholders More attention on training Absence of supplies Unavailability of drugs Restrict practice No counseling cards, chart booklets and recording forms Issues in purchase of medicines No supervision Expressed need for supervision Lack of district facilitators’ training Assessment of recording forms Weak referral system Lack of feedback Lack of transport Financial constraints Unavailable referral slips

## Factors supporting IMCI implementation

### Knowledge and perception about IMCI strategy

Most of the respondents from both groups were generally aware of IMCI strategy for the assessment and management of illness among children under 5 years of age with the aim to reduce under-five morbidity and mortality. Some of the respondents (trained physicians) even highlighted the five target diseases of the strategy (i.e. pneumonia, diarrhea, malnutrition, measles, and malaria). In addition, IMCI was considered by the majority of respondents as a ‘significant strategy’ which offers a holistic approach for the management of illness in children under 5 years of age.

According to the strategy we can holistically assess children … It is useful for us to know what to assess and monitor in children … we have to take history, ask mother, check the baby, weigh the baby etc … when to refer serious clients according to IMCI guideline. (Physician)

The strategy was perceived as ‘practical’ and ‘useful’ by most of the respondents at the PHC level. Besides having sufficient knowledge about the strategy, most of the respondents belonging to both groups also felt that IMCI is an important strategy for improving health outcomes in children. As stated by one of the respondents (District-level implementer) who described IMCI strategy as: ‘The best option for under five year children; can give good results’.

## Need for separate under-five clinic at PHC facilities

Having separate clinic for children under 5 years of age at PHC level emerged as one of the potential support factors toward IMCI implementation, this was identified by both groups of respondents. The respondents said that the PHC does not have a separate pediatric clinic and opined that a separate clinic would facilitate implementation of the strategy in a smooth way through allocation of adequate time, space, human resources, and required equipments and supplies. Separate under-five OPD was strongly supported by all respondents with a designated provider delegated with the responsibility for the case management of children under 5 years of age.

We must have separate under five clinic for children. One medical officer is responsible to look after both the general as well as under five clinics. If there is one designated provider in an isolated clinic, he will be able to give better attention for providing care to under five old children. (District-level implementer)

A few respondents (physicians) also said that they have been encouraged by district health management to run separate clinics for children under-five in compliance with IMCI protocol.

## Factors constraining IMCI implementation

### Lack of clear understanding of IMCI strategy

Although most of the respondents demonstrated a clear understanding of the strategy and its aims and objectives, some of the respondents at both district and provincial levels highlighted their lack of knowledge and awareness about the strategy: ‘It's a good program, but I don't know the objective of this strategy’ (District-level implementer).

It was also notable that some of the respondents even lacked awareness that IMCI is for children under five. ‘Objective of IMCI strategy is not clear to me. To me this strategy offers management of infants only’ (Provincial-level planner/policy maker).

A few respondents, in addition to acknowledging their lack of clear understanding of the strategy, emphasized the need for capacity building for improved monitoring of IMCI implementation. The following quote is illustrative: ‘I should be first trained in IMCI strategy … District management should be encouraged and trained so much that they can monitor IMCI implementation’ (District-level implementer).

## Lack of planning for IMCI implementation

Respondents (stakeholders) felt that there was a lack of planning at provincial and district health management levels for optimal implementation of the strategy. This was cited as one of the major impediments by the majority of the respondents. Respondents reported that planning was ignored to the extent that whenever any new project or program was introduced, there was emphasis on training without proper planning of activities: ‘Whenever any project comes, we do not discuss it we just start training – there is no planning at all …’ (Provincial-level planner/policy maker).

It is worth mentioning that lack of planning emerged as a concern equally among respondents at both provincial and district levels. Another group of respondents (trained physicians) also explicitly indicated ‘poor planning and lack of planning’ as a major hurdle for IMCI implementation. Some of the respondents even pointed out that a significant time lag existed between training and initiation of planning activities; for instance, despite capacity building workshops being initiated in 2004, it was only during the last few months prior to this study that district management decided to undertake planning for the implementation of IMCI strategy. A few of the respondents pointed out that the plan itself lacks clarity. When the reasons behind lack of planning were explored, ‘non-involvement of district pediatricians in IMCI implementation’ was identified as one of the major factors. Delayed and deficient IMCI planning being undertaken after capacity building served as one of the obstacles in progress toward sound implementation of the strategy. Lack of planning prior to capacity building was also reflected by ambiguous understanding in roles and responsibilities among key stakeholders.

## Ambiguity in roles and responsibilities toward IMCI implementation

In the context of expected roles and responsibilities for the IMCI strategy implementation, there was strong evidence of disparity among provincial- and district-level respondents. While most of the respondents at the provincial level were aware of their responsibilities, there was much uncertainty among district-level respondents toward their expected tasks for implementation of the strategy. When asked for the reasons, almost all district-level respondents held the MNCH program responsible for not providing detailed individual job responsibilities for the implementation. However, the respondents at the provincial level affirmed that in addition to the training of EDO-H from all districts, the official guidelines for the implementation of IMCI have been shared with key personnel at the district level. On the contrary, district-level stakeholders denied any support received in this regard. This clearly reflects a significant communication gap between district and provincial respondents.

I have not been involved from the higher level and have not being given any training. I have not received my job description by MNCH Program, I am not told what I can do, how to monitor IMCI implementation, still not clear what is my job description specific to strategy. (District-level implementer)

A few respondents at the district level even held the provincial MNCH program leadership responsible for their lack of direction and inability to effectively implement the strategy, possibly reflecting the communication gap between implementers and policy makers.

The ball is thrown in our court after providing IMCI training by MNCH program. I am helpless at this level …. I am helpless …. (District-level implementer)

## IMCI – a time-consuming task

Interviews with all the trained physicians revealed their interest, enthusiasm, and motivation to practice the strategy but the majority of them reported that IMCI practice are badly affected due to high out-patient flow in their clinics.

Yes I do practice IMCI after training. However, due to increase patient flow at health facility, I can't give proper time to all under five cases. (Physician)

Respondents also commented that ‘under five years old case management according to IMCI consumes lot of time’ which adversely affects their practice. The additional time for observing clients according to IMCI was cited as 7–20 min by respondents (trained physicians). Most of the respondents at both provincial and district levels had similar concerns and declared IMCI strategy as ‘problematic for doctors’ due to increased time spent in documentation, that is ‘filling case record forms’. However, one respondent differed in this opinion:

If doctors are well verse with IMCI strategy, then it would take 3–5 minutes to complete the assessment, provided they have adequate and conducive set up. (Provincial-level planner/policy maker)

## Emphasis on IMCI trainings vs. logistic support

Half of the stakeholders and most of the trained physicians in this study were of the opinion that the district health system's emphasis has been on training while ignoring the logistic aspect for implementing IMCI strategy. Equipments (including supplies, documentation, and reporting tools) and drugs were reported to be inadequate at PHC level and suggested to be available to trained doctors to practice the strategy. Most of the respondents cited unavailability of essential supplies as an obstacle toward proper implementation of the strategy: ‘There are just trainings going on … concept of translating the strategy into practice is not there’ (Provincial-level planner/policy maker).

A group of trained physicians talked about their inability to materialize training into practice with inadequate logistic support at PHC facilities cited as the major reason. The commonly cited supplies and reporting tools which were mentioned as unavailable included case record forms, mother's counseling cards, and IMCI chart booklets. Findings from the PHC survey also highlighted absence of counseling card and poor availability of chart booklet. However, case recording forms were found to be available in the majority of the surveyed facilities (recording forms for under 2 months were available in 56.3% and recording forms for 2 months to 5 years were available in 62.5% of the facilities).

When reasons for the unavailability and inadequacy of supplies and materials were explored, some respondents expressed a lack of defined responsibility in context of some of the tools. This interestingly links with their lack of defined roles and responsibilities as reported by the respondents and also reflects poor planning in context of strategy implementation.

Nobody in Government of Sindh actually realizes the importance of printing of case record form which is important for IMCI implementation. It has not being decided who will print it and this is specific to entire Pakistan. (Provincial-level planner/policy maker)

Similarly, a majority of the respondents disclosed the fact that the system does not have the provision of all IMCI drugs which also acts as a major hurdle toward the practice of IMCI strategy. In a few cases, unavailability of drugs for several months was also reported.

For the last 6-9 months we don't have drugs. The EDO-H was informed, but so far there has been no response. (Physician)

It came to light that physicians’ practice in accordance with the standard protocol of IMCI strategy has been affected because PHC facilities lacked required drugs according to WHO- and UNICEF-defined protocols. In this context, the facility survey also depicted shortage in availability of various IMCI drugs. These included, for instance, chloramphenicol (2 of 16 facilities), ampicillin or benzyl penicillin and gentamicin (3 of 16 facilities) and iron tablets and syrups (1 of 16 facilities), and so on. A few drugs such as cotrimoxazole, oral salbutamol, metronidazole, choloroquine, ringers lactate, and ORS were available in the majority of facilities. Use of substitute drugs for pyrantel pamoate was noted in the facility survey; however, doctors were found to be suspicious about their efficacy and efficiency. In addition, respondents were mostly unaware about the inadequacy and absence of pre-referral drugs which surfaced during the facility survey.

Respondents at PHC level were questioned regarding purchasing procedures for essential drugs to which one participant responded that despite having local purchase powers they are not allowed to buy drugs from outside. Unavailability of equipments, supplies, and drugs was perceived as creating a gap between physician's case management skills acquired during training and their practice of the strategy.

The trainings will be wasted because there is a big gap. For trained doctors, the system does not even have reporting forms and supplies. If you can't implement, you don't practice, you forget and you don't deliver. (Provincial-level planner/policy maker)

The above quote highlights an interconnected chain of obstacles in IMCI implementation. These obstacles ultimately stem from lack of sound planning resulting in unclear understanding of the strategy, ambiguity in roles and responsibilities among stakeholders, and emphasis toward skill development without the required logistic support through essential drugs and equipments.

## Lack of monitoring and supervisory mechanism

Supervisory and monitoring mechanism for observing the case management skills of trained physicians at PHC level was not reported by most of the respondents and was identified as a critical requirement for the successful implementation of IMCI strategy. Only a few respondents at PHC level stated that a focal person visited their facility only twice since they received IMCI training in 2007 to enquire about the progress of IMCI implementation and assessment of recording forms. Alarmingly, all respondents at RHCs, BHUs, and government dispensaries reported not receiving any supervision since their training and strongly emphasized the need of supervision and monitoring.

There should be supervisory visits, we must be asked what we did, how we did, whether we are implementing the strategy or not. (Physician)

The salient factor identified for the reported lack of supervision was the absence of capacity building of IMCI facilitators/trainers to observe the case management skills of trained doctors in the district. Lack of monitoring and supervisory mechanism for trained workforce possibly indicates lack of strategy planning and more emphasis on training rather than strengthening all aspects of health system.

## Inefficient referral system

In the context of referring sick children according to IMCI protocol, the referral system was perceived ‘weak’ and ‘inefficient’ by majority of the study respondents belonging from both groups; with lack of transport facilities in the district cited as the foremost concern.

Care takers of referred children do not go to the next level of care due to unavailability of transport at PHC. (Physician)

Provision of transport facilities for urgent referrals was emphasized by most of the respondents. In addition, financial constraints by the families of sick children also surfaced as an impediment to optimal referrals because families, rather than going to tertiary care hospital or any referred care facility, go home and arrange money for transport and anticipated expenses for the treatment of their child. Furthermore, feedback from the referred care facility was also cited as ‘being non-existent at the district.’ In this regard, respondents at PHC level felt interested in getting feedback from the referred care facility for every referral. Moreover, on enquiring about the availability of referral slips/forms at the PHC, a few of the respondents at PHC reported the use of plain paper for referral as a substitute for a referral form, due to its unavailability. However, referral forms were found to be available in the majority (10 out of 16) of the surveyed facilities.

## Discussion

Study findings revealed that IMCI implementation in Matiari district has been constrained by numerous factors. However, a few supporting factors were also identified, including knowledge and perception regarding the effectiveness of IMCI strategy by the majority of the IDI respondents and a perceived need for a separate under-five clinic for children. The latter reflects their confidence in the strategy and emerges as a major perceived support factor which has not yet been reported from other studies.

Among the constraint factors for implementation, findings drew attention toward inappropriate or insufficient planning by the stakeholders involved. IMCI implementation planning is crucial because it provides the impetus for key MoH personnel to understand the major steps of overall implementation and also sets direction for implementation at the district level (28). WHO-IMCI planning guide assists countries to undertake IMCI strategy by describing the phased approach for planning and implementing interventions for the strategy (29). It initiates early implementation by a national-level working group which involves districts in planning, reviewing the early implementation phase, and planning for expansion. District staff needs to be made familiar with IMCI guidelines by national working groups prior to undertaking district-level IMCI planning workshops (29). Later, major responsibility lies with districts to initiate planning for IMCI implementation by getting support from the provincial level if needed. Table 7 lists some of the recommended aspects needed for district-level planning of IMCI implementation.

**Table 7:** District planning for IMCI implementation (29)

Improvement in skills of health staff IMCI training courses Follow-up visits after training How to build district training capacity Improvement of health workers’ skills at referral level Improvement in the health system Availability of the drugs needed for IMCI at facilities Organization of work at health facilities Improving referral pathways and services Supervision Linking IMCI classifications and the HIS Improvement in family and community practices Documentation of early implementation District budget

In our study, lack of planning emerged as a critical impediment to adequate implementation. It was found that besides poor planning, the objectives of the strategy were unclear to a few stakeholders at both district and provincial levels, reflecting a need for optimal capacity building in IMCI strategy. In this regard, WHO strongly emphasizes the exposure of key personnel in provincial MoH to IMCI training, planning, and adaptation procedures (28), which was not found in Matiari district.

Some key personnel at district and provincial levels were unaware of their expected roles and responsibilities in the implementation process. This is extremely alarming and questions the future successful implementation of the strategy in the district. The discrepancies among provincial- and district-level respondents toward the provision of detailed guidelines for IMCI implementation highlights the communication gap among key personnel involved in IMCI implementation. In this regard, the provincial MNCH program was also held responsible by some of the respondents by virtue of its mandate to support districts in the implementation of IMCI strategy by providing all the required logistic support (30).

Findings indicated inadequate district health care system preparedness for the implementation of the strategy which is exhibited by lack of IMCI implementation planning as an underlying cause leading to lack of defined responsibilities toward implementation. Its manifestations later surfaced in the form of inadequate district health care management support for trained doctors to practice the strategy. This includes shortage of supplies and drugs, lack of monitoring and supervisory mechanisms, and a weak referral system. Training of health care personnel in IMCI strategy is an important component for successful implementation (31). The respondents in our study did not undermine the importance of training but highlighted the significance of provision of essential supplies and drugs. Respondents argued that the latter would facilitate the trained personnel to implement their training in real settings and the training *per se* would not be able to achieve the results. Studies from countries have shown mixed results over the availability of required logistics for implementation support. A study in China demonstrated increased availability of essential IMCI drugs, equipments, and supplies with IMCI implementation at the health care facilities (7). Contrary to this, in a few countries, IMCI drugs are not part of the national essential drug list (12). Inadequate availability of drugs and equipments was also evident in our study. In this context, district management was held accountable for the provision of some of the equipments. However, due to a lack of clarity regarding expected responsibilities among key district and provincial stakeholders, the responsibility of providing supplies and equipments lies with the provincial MoH. Furthermore, use of IMCI substitute drugs at PHC facilities calls for a review by a representative from the WHO and UNICEF at country level to ensure effectiveness of the substitute treatment.

The study has identified weak referral system as one of the important constraints. IMCI multi-country evaluation has also pointed out inadequate referral systems with lack of referral facilities in various countries (8). In our study, non-availability of transport was quoted as the major reason for a weak referral system. Another important weakness of the referral system is non-availability of pre-referral drugs as demonstrated by our findings. Similar findings have been reported from other countries as well (5). Moreover, drugs for urgent referral were reported as absent from all facilities. Alarmingly, none of the respondents mentioned the inadequacy of pre-referral drugs at PHCs which was identified during the facility survey. This calls for a critical review of training components so that trained personnel appreciate the significance of having lifesaving pre-referral drugs available at PHC facilities.

Generally, the district supervisory and monitoring mechanism was not in compliance with WHO protocols, which recommend a follow-up visit to all trained health workers within 4 to 6 weeks of completion of training (32). Similarly, various studies in the past have also highlighted the significance of supervisory visits and follow-ups for trained manpower after training (33, 34). The underlying reason identified for the lack of a supervisory and monitoring mechanism in the district was the deficient pool of trained supervisors.

The lack of district health care delivery system preparedness for IMCI implementation has implications to reduce the overall compliance to IMCI protocol by a trained health workforce and can significantly affect the quality of care for sick children. Similarly, studies have evidenced that inadequate health care system support resulted in poor health workers’ performance (10, 34). Study findings highlight the need for the provincial MNCH program to strengthen district health care system support by building their capacity in planning and implementation of the strategy especially in the post-devolution scenario in Pakistan (14). The role of district implementation planning is crucial in setting the stage for planning for improvement in the overall health system by assuring the availability of drugs and supplies, supervisory systems and plans for strengthening the referral system (29).

Overall, our study findings have demonstrated that Matiari district is ill equipped for IMCI implementation. The study also exposes the interdependencies of the constraining factors, such as lack of sound planning resulting in unclear understanding of the strategy; leading to ambiguous roles and responsibilities among stakeholders which manifest as inadequate availability of supplies and drugs at PHC facilities. Addressing these barriers is likely to have a cumulative effect on facilitating IMCI implementation. We therefore recommend that policy makers at the provincial level should undertake a participatory planning exercise involving the maximum number of stakeholders at district level. This planning exercise should take into account the gaps identified by this study including a stronger focus on improved monitoring and supervision of trained staff, provision of supplies and logistic support, and a clear enunciation of stakeholder roles and responsibilities. The mandate for IMCI adoption by districts falls within the purview of the provincial MNCH program. We, therefore, recommend that the provincial MNCH program should take the lead in this exercise, bringing all relevant stakeholders to the planning forum, strengthening links between provincial- and district-level stakeholders. The MNCH program should also take steps aimed at ensuring the availability of the required budget for the procurement of essential IMCI drugs and supplies for the district, availability of transportation services in the district, and also to support the district in monitoring and evaluation of IMCI implementation by building their capacity. All these recommendations are in line with a project cycle document developed under federal MoH prior to devolution (30). The gap seems to lie in execution of these plans. Exploring reasons/barriers for effective implementation of the plans can be the future research avenues, which can bring meaningful insight to policy makers and stakeholders for IMCI implementation.

These recommendations gain importance in Pakistan's post-devolution scenario, whereby the federal MoH has been devolved to the provinces under a constitutional amendment (14). The provincial MoH now enjoys greater autonomy with little change in human resource and service delivery infrastructure, thereby paving the way for improved planning with greater involvement of all stakeholders’ right down to the grassroots level. Currently, the federal government is providing support to provincial governments in implementing programs under the devolved system, including financial and technical support, ensuring sufficient resources for implementation. This also implies greater accountability of the provincial MoH and the need for improved logistic support to ensure IMCI implementation. Beside this, the provinces are currently in process of formulating their project cycle document; the results of the study would be crucial in facilitating the development of logical frameworks which can serve as a sound planning approach for IMCI implementation.

All key stakeholders at provincial level interviewed in this study also serve as the key implementers for other districts and our findings could serve as the baseline for Provincial MoH Sindh to evaluate the capacity of other districts in Sindh toward effective IMCI implementation. Moreover, MoH also needs to assess and address the gaps in the planning and implementation capacity of key provincial stakeholders.

## Reliability, transferability, and dependability

Reliability in a qualitative study is achieved by explaining the methodological framework and analytical constructs (35). In the context of this study, several steps were taken to ensure reliability: an embedded design was chosen; separate IDIs guides was developed for trained physicians and policy makers/implementers; participants were purposively selected, that is those who are expected to play a key role in implementation (trained physicians and district- and provincial-level stakeholders); interviews were conducted in respondents language of choice (three out of eight stakeholders’ interviews were conducted in English, while the remaining were conducted in Urdu). All interviews were transcribed verbatim and translated into English and finally, the first author verified the transcriptions by listening to the audio tapes twice.

Quantitative part included a survey of PHC facilities in the district using the WHO facility survey checklist. Triangulation was carried out via use of two different groups of informants and primary health facility survey to verify findings related to logistics support.

In context of reliability in facility survey, findings obtained from the facility survey remained consistent over a month during the course of the study as demonstrated by random checks by the first author. Half of PHC facilities were surveyed by the first author and the other half by a trained data collector under supervision of the first author.

For analytical constructs, three of the authors (N. P., N. R., and N. S.) agreed in the way the codes and categories were labeled and categorized, which were reviewed by the last author (X. G.). In the later stages, two broad themes were identified in consultation with all authors. However, proportions were calculated from the findings obtained from PHC facility survey. Transferability refers to the extent to which the findings can be transferred to other settings (25). For transferability, description of context, data collection methods, selection of participants, and analysis is given with rich presentation of findings in appropriate quotations (25). Findings of the study can be transferred to other districts of the Sindh which share similar socio-demographic and management characteristics. To facilitate transferability, clear description of context, selection of participants, data collection, and process of analysis has been given with presentation of findings in quotations. Dependability also deals with detailed description of processes within the study, thereby enabling future researches to repeat the work if necessary (36). This has also been addressed with provision of detailed methodology in the paper.

For achieving validity in facility survey, WHO's facility survey tool on ‘Equipment and supply checklist’ was adapted and modified in context of including specific items (first- and second-line antibiotics and list of required supplies) mentioned in the IMCI training guide (37). Furthermore, to ensure content validity tool was assessed by co-authors to check the aspects it covers (38).

## Strengths and limitations

Our study provides crucial insight into the complexities of implementation issues prevailing not only at the PHC facility level but also at district and provincial levels, 7 years into implementation. Findings also facilitate our understanding of the root causes of inadequate IMCI implementation with unpreparedness for the strategy exhibited by planners (provincial level) and implementers (district level). Another major strength of the study was the embedded facility survey in the qualitative design which validated the findings concerning inadequate health care system support for the required logistics.

One of the limitations of the study was the limited time available for IDIs from few of the trained physicians due to high patient flow at outpatient clinics. Another shortcoming of the study was its limitation to explore only supply side factors concerning facility-based IMCI implementation and hence user's (community) perspective was beyond the scope of this study. Community IMCI (C-IMCI), which is the third component of IMCI, seeks to strengthen the linkage between health services and communities and to improve family and community practices through community health workers (39). Although a pool of lady health workers (LHWs) has been trained in C-IMCI in the country (17), it is yet to be implemented in the study area and hence was not included in the study. Future IMCI implementation and evaluation research should be conducted while taking into account both supply and demand side factors; cutting across all three components of IMCI strategy which can provide a holistic picture of IMCI implementation. Moreover, time constraints experienced at the facility during IDIs with few physicians due to high patient flow could possibly be dealt with by scheduling follow-up interviews with physicians after clinic hours based on their availability.

## Conclusion

This research illustrates the lack of district health care system preparedness toward IMCI implementation. Our findings indicate that the district has sound implementation potential as reflected by respondent's perception regarding effectiveness of IMCI strategy. However, bottlenecks at health care facility and at health care management level have constrained the implementation process in Matiari district. Findings from this study give further credence to the information reported during an early implementation phase in the country concerning lack of health care system support toward IMCI implementation (19). This indicates that our health system has not evolved in terms of lessons learned from the past and from other developing countries’ experience in IMCI implementation. Within the context of devolution of the MoH to the provinces and Pakistan's impending failure to meet Millennium Development Goal No. 4 of reducing child mortality (40) the need for sound planning, training, supervision, and logistic support becomes even more important. This study recommends that the provincial MoH and MNCH program need to develop synergy and to assess the situation to streamline IMCI implementation in the district.

## Appendix 1

**Table:**

Interview guide for physicians (Abridged version) What is your understanding of IMCI strategy? Objective of the strategy Significance of the strategy In your experience, which factors influence application of IMCI guidelines in managing under-five cases at your work place? Support received Constraints observed In your experience, what problems are faced by physicians while referring under-five cases according to IMCI guidelines? How do you perceive the role and significance of monitoring and supervision for trained IMCI doctors working at PHC facilities? In your opinion, how can you better implement IMCI strategy at your work place? Recommendations for the effective implementation of IMCI at first level care facilities
Interview guide for policy makers/planners & implementers (Abridged version) What is your understanding of IMCI strategy? Objective of the strategy Significance of the strategy What roles do you play in your current designation for implementation of IMCI strategy at public-sector primary health care facilities in Matiari district? In your best judgment, what factors influence IMCI implementation at public-sector PHC facilities in Matiari district? Supports for implementation Constraints influencing implementation In your opinion, what is the role and significance of monitoring and supervision of trained IMCI doctors working at PHC facilities in ensuring effective IMCI implementation? In your view what mechanisms are in place for monitoring and supervision for trained IMCI doctors in Matiari? In your view, what policy and planning initiatives have been taken in Matiari district to ensure effective IMCI implementation? What policy-level initiatives would you recommend for effective implementation of IMCI strategy at public-sector PHC facilities in Matiari?

## References

[1] Powell J (1997). Improving child health.

[2] Anthony D, Mullerbeck E Committing to child survival: a promised renewed. Progress Report 2012.

[3] Maternal, Newborn, Child and Adolescent Health. Integrated Management of Childhood Illness (IMCI) World Health Organization. http://www.who.int/maternal_child_adolescent/topics/child/imci/en/.

[4] Ahmed HM, Mitchell M, Hedt B (2010). National implementation of Integrated Management of Childhood Illness (IMCI): policy constraints and strategies. Health Policy.

[5] Amaral J, Gouws E, Bryce J, Leite AJ, Cunha AL, Victora CG (2004). Effect of Integrated Management of Childhood Illness (IMCI) on health worker performance in Northeast-Brazil. Cad Saude Publica.

[6] Chopra M, Patel S, Cloete K, Sanders D, Peterson S (2005). Effect of an IMCI intervention on quality of care across four districts in Cape Town, South Africa. Arch Dis Child.

[7] Zhang Y, Dai Y, Zhang S (2007). Impact of implementation of Integrated Management of Childhood Illness on improvement of health system in China. J Pediatr Child Health.

[8] Department of Child and Adolescent Health and Development, World Health Organization (2002) The multi-country evaluation of IMCI effectiveness cost and impact (MCE).

[9] Arifeen SE, Bryce J, Gouws E, Baqui AH, Black RE, Hoque DM (2005). Quality of care for under-fives in first-level health facilities in one district of Bangladesh. Bull World Health Organ.

[10] Huicho L, Davila M, Campos M, Drasbek C, Bryce J, Victora CG (2005). Scaling up Integrated Management of Childhood Illness to the national level: achievements and challenges in Peru. Health Policy Plan.

[11] World Health Organization (2004). The analytic review of the integrated management of childhood illness strategy. Final Report Nov 2003.

[12] Goga AE, Muhe LM (2011). Global challenges with scale-up of the integrated management of childhood illness strategy: results of a multi-country survey. BMC Public Health.

[13] World Fact Book for Pakistan http://www.cia.gov/library/publications/the-world-factbook/geos/pk.html.

[14] An analysis. Health and the eighteenth constitutional amendment (2011). http://www.pildat.org/Publications/publication/publichealth/PILDATAnalysis-Healthandthe18thConstitutionalAmendments.pdf.

[15] Regional Health System Observatory, World Health Organization (2007). Health system profile Pakistan. Cairo, Egypt. http://hinfo.humaninfo.ro/gsdl/healthtechdocs/en/m/abstract/Js17305e/.

[16] National Institute of Population Studies (NIPS) [Pakistan], Macro International (2008). Pakistan demographic and health survey 2006–07.

[17] World Health Organization Integrated Management of Childhood Illness (IMCI) in Pakistan. http://www.who.pak.org/index.php?option=com_rubberdoc.

[18] Nisar N (2003). Integrated management of childhood illness and health system reforms in Pakistan. J Pakistan Med Assoc.

[19] Associate AR (2002). Evaluation report. Process evaluation of early implementation phase of IMCI in two pilot districts of Pakistan. UNICEF. http://www.unicef.org/spanish/evaldatabase/index_14601.html.

[20] Child Health and Development, World Health Organization Implementation of IMCI in Pakistan. http://www.emro.who.int/child-health/strategy-implementation/implementation-of-imci-in-pakistan.html.

[21] Creswell JW, Clark VLP (2011). Designing and conducting mixed methods research.

[22] Pirzada A

[23] Grady MP (1998). A qualitative and action research: a practitioner handbook.’ Bloomington. Phi Delta Kappa Educational Foundation.

[24] Department of Child and Adolescent Health and Development (2003). Health facility survey. http://www.who.int/maternal_child_adolescent/documents/9241545860/en/index.html.

[25] Graneheim UH, Lundman B (2004). Qualitative content analysis in nursing research: concepts, procedures and measures to achieve trustworthiness. Nurse Educ Today.

[26] Hsieh H-F, Shannon SE (2005). Three approaches to qualitative content analysis. Qual Health Res.

[27] Babbie E (1992). The practice of social research.

[28] Lambrechts T, Bryce J, Orinda V (1999). Integrated Management of Childhood Illness: a summary of first experiences. Bull World Health Organ.

[29] Department of Child and Adolescent Health and Development, WHO IMCI Planning Guide. Gaining experience with the IMCI strategy in a country.

[30] Ministry of Health, Government of Pakistan (2006). PC 1. National Maternal Newborn and Child Health (MNCH) Program. 2006–2012. http://www.pc.gov.pk/downloads/pc1-forms_vertical-health-program/National%20Maternal%20Newborn%20and%20Child%20Health%20%20Programme.pdf.

[31] Bishai D, Mirchandani G, Pariyo G, Burnham G, Black R (2008). The cost of quality improvements due to Integrated Management of Childhood Illness (IMCI) in Uganda. Health Econ.

[32] Department of Child and Adolescent Health and Development, World Health Organization (1999). Guidelines for follow-up after training. WHO/UNICEF course on Integrated Management of Childhood Illness for first-level health workers Supervisor's guide. http://www.union-imdp.org/files/resources/training/Integrated%20management%20of%20childhood%20illness%20for%20first-level%20health%20workers.pdf.

[33] Goga AE, Muhe LM, Forsyth K, Chopra M, Aboubaker S, Martines J (2009). Results of a multi-country exploratory survey of approaches and methods for IMCI case management training. Health Res Policy Syst.

[34] Pariyo GW, Gouws E, Bryce J, Burnham G (2005). Improving facility-based care for sick children in Uganda: training is not enough. Health Policy Plan.

[35] Ali AM, Yusof H (2011). Quality in qualitative studies: the case of validity, reliability and generalizability. Issues Soc Environ Account.

[36] Shenton AK (2004). Strategies for ensuring trustworthiness in qualitative research projects. Educ Inform.

[37] Maternal, Newborn, Child and Adolescent Health, World Health Organization IMCI chart booklet. http://www.who.int/maternal_child_adolescent/documents/IMCI_chartbooklet/en/.

[38] Twycross A, Shields L (2004). Validity and reliability – what's it all about? Part 1 validity in quantitative studies. Paediatr Nurs.

[39] World Health Organization (2004). Child health in the community. Community IMCI. Briefing package for facilitators.

[40] Balck RE, Cousens S, Johson HL, Lawn JE, Rudan I, Bassani DG (2010). Global, regional and national casuses of child mortality in 2008: a systematic analysis. Lancet.

